# Corticofugal VIP Gabaergic Projection Neurons in the Mouse Auditory and Motor Cortex

**DOI:** 10.3389/fncir.2021.714780

**Published:** 2021-07-23

**Authors:** Alice Bertero, Charles Garcia, Alfonso junior Apicella

**Affiliations:** Department of Biology, Neurosciences Institute, University of Texas at San Antonio, San Antonio, TX, United States

**Keywords:** long-range, GABAergic, VIP, Som, Parv, auditory cortex, motor cortex

## Abstract

Anatomical and physiological studies have described the cortex as a six-layer structure that receives, elaborates, and sends out information exclusively as excitatory output to cortical and subcortical regions. This concept has increasingly been challenged by several anatomical and functional studies that showed that direct inhibitory cortical outputs are also a common feature of the sensory and motor cortices. Similar to their excitatory counterparts, subsets of Somatostatin- and Parvalbumin-expressing neurons have been shown to innervate distal targets like the sensory and motor striatum and the contralateral cortex. However, no evidence of long-range VIP-expressing neurons, the third major class of GABAergic cortical inhibitory neurons, has been shown in such cortical regions. Here, using anatomical anterograde and retrograde viral tracing, we tested the hypothesis that VIP-expressing neurons of the mouse auditory and motor cortices can also send long-range projections to cortical and subcortical areas. We were able to demonstrate, for the first time, that VIP-expressing neurons of the auditory cortex can reach not only the contralateral auditory cortex and the ipsilateral striatum and amygdala, as shown for Somatostatin- and Parvalbumin-expressing long-range neurons, but also the medial geniculate body and both superior and inferior colliculus. We also demonstrate that VIP-expressing neurons of the motor cortex send long-range GABAergic projections to the dorsal striatum and contralateral cortex. Because of its presence in two such disparate cortical areas, this would suggest that the long-range VIP projection is likely a general feature of the cortex’s network.

## Introduction

The auditory cortex is a six-layer structure characterized by different cell types. In a very simplistic way, these neurons, according to the neurotransmitter they release, can be divide into two main groups: glutamatergic/excitatory pyramidal neurons (about 70–80%) and GABAergic/inhibitory neurons (about 20–30%). Moreover, this neuronal subdivision of the cortex leads to the overall principle of cortical organization that excitation is both local and long-range, while inhibition is described as being exclusively local (for review, see Isaacson and Scanziani, 2011). However, the existence of long-range GABAergic neurons in rats, cats, and monkeys has been reported anatomically since the 80s (Seress and Ribak, 1983; Ribak et al., 1986; Toth and Freund, 1992; McDonald and Burkhalter, 1993; Toth et al., 1993; Tomioka et al., 2005, 2015; Higo et al., 2007, 2009; Tomioka and Rockland, 2007; Caputi et al., 2013). Only recent investigation has been engaged to understand how different subtypes of long-range GABAergic projections play distinct roles in cortical processing according to their differences in anatomical, electrophysiological, molecular content, and synaptic connectivity patterns (Melzer et al., 2012, 2017; Lee et al., 2014; Rock and Apicella, 2015; Rock et al., 2016, 2018; Zurita et al., 2018a; Bertero et al., 2019, 2020). Our lab showed that both Parvalbumin- and Somatostatin-expressing neurons in the sensory and motor cortices provide direct inhibitory projection to multiple cortical and subcortical areas (Rock et al., 2016, 2018; Zurita et al., 2018a; Bertero et al., 2019, 2020). However, no long-range GABAergic VIP-expressing neurons from these cortical regions have been shown yet. We addressed this fundamental question using anterograde and retrograde anatomical methods. Using these techniques, we demonstrate, for the first time, the existence of VIP-expressing GABAergic neurons in the auditory and motor cortices with cortico-cortical and cortico-subcortical long-range GABAergic projections.

## Materials and Methods

### Animals

All animal procedures were approved by the Institutional Animal Care and Use. Procedures followed animal welfare guidelines set by the National Institutes of Health. Mice were housed in a vivarium maintaining a 12 h light/dark schedule and given ad libitum access to mouse chow and water. Male and female homozygous VIP-Cre mice were used in this study and were injected at postnatal day 35: B6J.Cg-Viptm1(cre)Zjh/AreckJ (the Jackson Laboratory, stock number 031628). Auditory Cortex injections (anterograde): *N* = 5 mice from 3 litters; Motor cortex injections (anterograde): *N* = 3 mice from 2 litters; Auditory Cortex injections (retrograde): *N* = 3 mice from 2 litters.

### Stereotaxic Injections

As described in our previous studies mice were initially anesthetized with isoflurane (3%; 1 L/min O_2_ flow) and head-fixed on a stereotaxic frame (Model 1900; Kopf Instruments) using non-rupture ear bars. Anesthesia was maintained at 1–1.5% isoflurane for the duration of the surgery. A warming pad was used to maintain body temperature during the procedure. Standard aseptic technique was followed for all surgical procedures. Injections were performed using a pressure injector (Nanoject II; Drummond Scientific) mounted on the stereotaxic frame. Injections were delivered through a borosilicate glass injection pipette (Wiretrol II; Drummond Scientific) with a taper length of ~30 mm and a tip diameter of ~50 μm. The pipette remained in place for 5 min before starting injecting at 1 nl/s rate, 15 s waiting period after each nl, and was left in place for 5 min after the injection to avoid viral backflow along the injection tract. Both male and female mice, P35–40 at the time of the injection, were utilized in these experiments. Viral preparation used in this study: AAV1-Syn-Flex-ChrimsonR-tdTomato (AAV-flex-ChRimson-tdTomato, titer 4.1 × 10^12^ GC/ml (UNC Gene Therapy Center Vector Core); AAV1-CAG-Flex-EGFP-WPRE, titer 3.1 × 10^13^ VG/ml (AAV1-flex-GFP, Addgene viral prep #51502-AAV1). Coordinates for injections for the right auditory cortex: 2.6 mm posterior and 4.5 mm lateral from Bregma, 0.9 mm below the brain’s surface. Coordinates for injections for right motor cortex: 1 mm posterior and 1.7 mm lateral from Bregma, 0.9 mm below the brain’s surface.

### Immunohistochemistry

Four to Five weeks after injection, mice were anesthetized with isoflurane and decapitated. Coronal slices (200 or 300 μm thick) were sectioned on a vibratome (VT1200S; Leica) in a chilled cutting solution containing the following (in mM): 100 choline chloride, 25 NaHCO_3_, 25 D-glucose, 11.6 sodium ascorbate, 7 MgSO_4_, 3.1 sodium pyruvate, 2.5 KCl, 1.25 NaH_2_ PO_4_, 0.5 CaCl_2_. Slices were then imaged acutely on an Olympus Macroscope with an appropriate RFP filter at a 1.6×, 5×, or 6.4× magnification lens. Slices were then postfixed for 3 h at room temperature in PBS buffered 1% PFA solution with gentle shaking. For retrograde experiments, 2 weeks after injection, mice were transcardially perfused with 4% PFA in Phosphate Buffered Saline (PBS), the brain was extracted and postfixed overnight in 4% PFA solution and sliced with a vibratome at 100 μm thick coronal slices.

Immunohistochemical procedures were performed on free-floating sections using rabbit anti-RFP (for tdTomato, 1:500, Abcam, cat #ab62341) or Chicken-anti-GFP (1:1,000; Abcam, ab13970) primary antibody, followed by Alexa Fluor 594 goat anti-rabbit IgG (1:500, Life Technologies) or AlexaFluor 488 goat anti-chicken IgG (1:500; Life Technologies) secondary antibody. Briefly, slices were washed 3–6 times in PBS 0.3% Triton X-100, and then incubated 1 h at room temperature with blocking solution (PBS with 0.3% Triton X-100 and 5% Goat serum), and overnight at 4°C with primary antibody solution with gentle shaking. After 16–24 h, slices were thoroughly washed with PBS containing 0.3% Triton X-100 at least three times for at least 10 min each wash, and then incubated 2 h at room temperature with secondary antibody solution (blocking solution with the appropriate combination of secondary antibodies). Before mounting, the slices were washed again at least three times with PBS containing 0.3% Triton X-100 for at least 10 min each wash and then mounted in Fluoromount-G (Southern Biotech). Confocal images were taken with a Zeiss LSM-710 microscope at 10×, 20×, 40× magnifications with an appropriate filter set for AlexaFluor-488/594.

Image adjustment was performed with ImageJ (National Institutes of Health) or Adobe Photoshop for brightness/contrast corrections and pseudo coloring. Atlas reference tables were modified from the Allen Brain Atlas.

Quantification of laminar distribution of GFP-expressing VIP neurons was performed in the injection site of VIP-Cre mice injected with AAV-flex-GFP in the right auditory cortex and used for retro experiments. 100 μm thick slices (*n* = 12 slices from two mice, one litter) were acquired at 4× magnification, and a normalized 50 μm spaced grid was superimposed to cover the whole cortical thickness from Pia (0 μm) to white matter (1 mm). GFP-expressing cells were counted using the Cell Counter plugin of ImageJ.

### Slice Preparation and Recording

Mice were anesthetized with isoflurane and decapitated. Coronal slices (300 μm) containing the injection site were sectioned on a vibratome (VT1200S; Leica) in a chilled cutting solution containing the following (in mM): 100 choline chloride, 25 NaHCO_3_, 25 D-glucose, 11.6 sodium ascorbate, 7 MgSO_4_, 3.1 sodium pyruvate, 2.5 KCl, 1.25 NaH_2_PO_4_, 0.5 CaCl_2_. These slices were incubated in oxygenated artificial cerebrospinal fluid (ACSF) in a submerged chamber at 35–37°C for 30 min and then room temperature (21–25°C) until recordings were performed. ACSF contained the following (in mM): 126 NaCl, 26 NaHCO_3_, 10 D-glucose, 2.5 KCl, 2 CaCl_2_, 1.25 NaH_2_PO_4_, 1 MgCl_2_; osmolarity was ~290 Osm/L. Cell-attached and whole-cell recordings were performed in 31–33°C ACSF. Thin-walled borosilicate glass pipettes (Warner Instruments) were pulled on a vertical pipette puller (PC-10; Narishige) and typically were in the range of 3–5 MΩ resistance when filled with a potassium-based intracellular solution containing the following (in mM): 120 potassium gluconate, 20 KCl, 10 HEPES, 10 phosphocreatine, 4 ATP, 0.3 GTP, 0.2 EGTA, and 0.3–0.5% biocytin). Signals were sampled at 10 kHz and filtered at 4 kHz. Hardware control and data acquisition were performed by Ephus^1^ (Suter et al., 2010). ChRimson positive VIP neurons in the injection site were patched and photostimulated in cell-attach: recordings were coupled with photostimulation with 615 nm wavelength red LED (CoolLED pE excitation system) and 60× water-immersion objective at 0.2 and 0.4 mW/mm2 LED power. During whole-cell recordings, neurons were filled with an internal solution containing 0.3–0.5% biocytin. Filled neurons were held for at least 20 min, and then the slices were fixed in a formalin solution (neutral buffered, 10% solution; Sigma-Aldrich) for 1–5 days at 4°C. The slices were washed in PBS (six times, 10 min per wash) and placed in a 4% streptavidin-AlexaFluor-488 conjugated (Life Technologies) solution, incubated at 4°C overnight, washed in PBS (six times, 10 min per wash), and mounted with Fluoromount-G (SouthernBiotech) on a glass microscope slide. Confocal images were taken with a Zeiss LSM-710 microscope at 40× magnifications. Image adjustment was performed in ImageJ (National Institutes of Health) for brightness/contrast corrections and pseudocoloring. Neurons were morphologically reconstructed in three dimensions using the Simple Neuritre Tracer plugin on ImageJ software (Schindelin et al., 2012).

## Results

**Figure 1:**
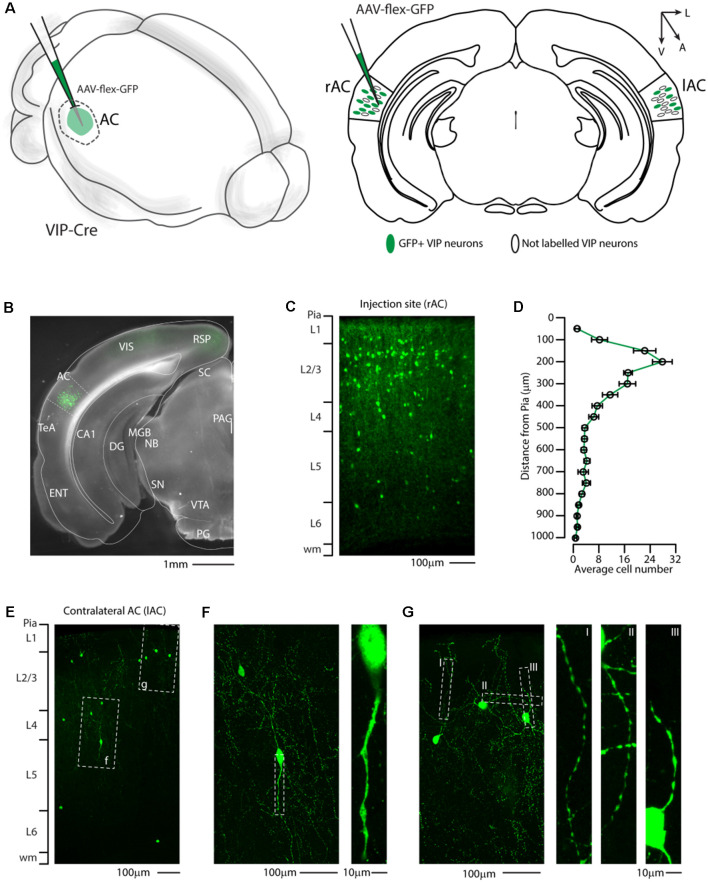
Distribution of VIP-expressing neurons in the auditory cortex. **(A)** Schematic representation of right auditory cortex viral injection of AAV-flex-GFP. VIP-expressing neurons in both the injected (right AC, rAC) and contralateral (left, lAC) auditory cortex express GFP in a Cre-dependent manner. **(B)** Representative coronal section of an acute slice of the injection site (200 μm thick), with bright field (gray) and GFP expressing neurons (green). The Allen brain atlas coronal table superimposed for reference indicates the correct targeting of the auditory cortex. Scale bar: 1 mm. **(C)** High magnification confocal image of the injection site showing GFP expressing neurons in the right auditory cortex. Scale bars: 100 μm. **(D)** Laminar distribution of VIP neurons in the auditory cortex, quantified every 50 μm from Pia (0) to white matter (1 mm). Data are expressed ad mean ± s.e.m. **(E)** Representative high magnification confocal image of the contralateral Auditory cortex showing retrograde labeled GFP expressing neurons. Scale bars: 100 μm. **(F,G)** Details of dashed square (f and g) of **(E)**, with corresponding high magnification of the dendritic arborization of GFP+ VIP-expressing neurons showing their characteristic thin and aspiny morphology. Scale bars: 100 μm and 10 μm. RSP, retrosplenial cortex; VIS, visual cortex; TeA, temporal association cortex; AC, auditory cortex; ENT, enthorinal cortex; CA1, Cornu Ammonis of the hippocampus subfield 1; DG, dentate gyrus; SC, superior colliculus; MGB, medial geniculate body; NB, nucleus of the brachium of the inferior colliculus; PAG, periacqueductal gray; SN, substantia nigra; VTA, ventrotegmental area; PG, pontine gray.

First, we injected a cre-dependent AAV virus expressing a cytoplasmic reporter (AAV-flex-GFP) in the right auditory cortex of VIP-Cre mice (Figures 1A,B) and determined the laminar distribution of GFP positive neurons. This allowed us to clearly identify GFP+/VIP-Cre soma in the auditory cortex (Figures 1B,C) and their characteristic distributions among cortical layers, with a peak in layer 1 and 2/3 (Figure 1D), as already described in genetically labeled VIP neurons (Mesik et al., 2015; Pronneke et al., 2015). Interestingly, since our lab and others (Rothermel et al., 2013; Rock et al., 2016, 2018; Zurita et al., 2018a; Bertero et al., 2019, 2020) have described that AAV1-Flex viral vectors exhibited both anterograde and retrograde transfection capabilities, we checked the contralateral auditory cortex and found GFP-labeled neurons (Figure 1E). The retrograde labeled neuron’s bipolar morphology and aspiny dendritic arborization are compatible with VIP-expressing neurons (Figures 1F,G). This experiment supports the hypothesis that not only Parv- and Som-expressing neurons (Rock et al., 2016, 2018; Zurita et al., 2018a; Bertero et al., 2019, 2020), but also VIP-expressing neurons send long-range projections from the auditory cortex.

**Figure 2:**
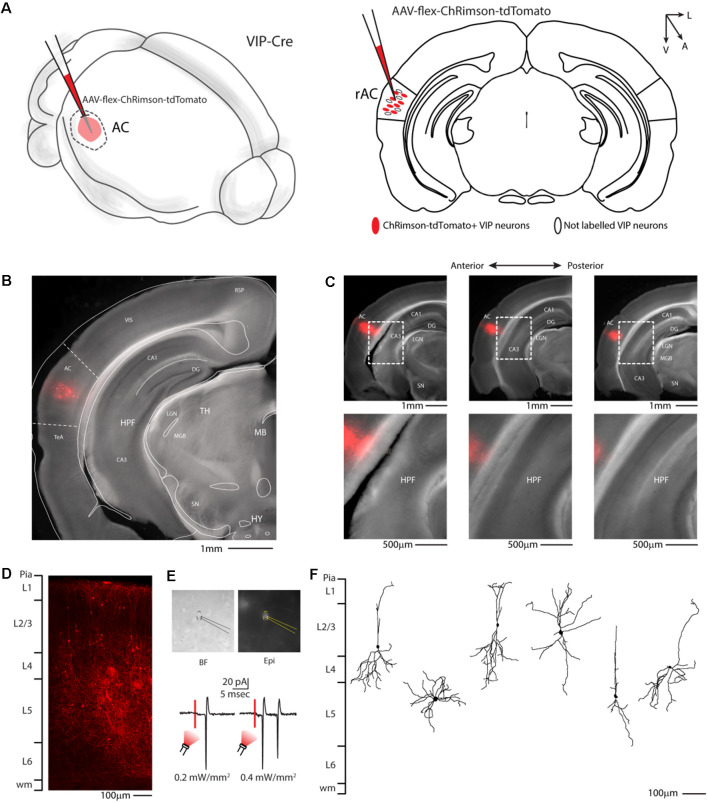
Auditory cortex injection of AAV-flex-ChRimson-tdTomato. **(A)** Schematic representation of right auditory cortex viral injection. **(B)** Representative coronal section of an acute slice of the injection site (200 μm thick), with bright field (gray) and tdTomato expressing neurons (red). The Allen brain atlas coronal table superimposed for reference indicates the correct targeting of the Auditory cortex. Scale bar: 1 mm. **(C)** Top panel: antero-posterior and dorsal ventral spread of ChRimson-tdTomato injection in AC. Scale Bars: 1 mm. Bottom panel: high magnification image of the injection site showing no evidence of ChRimson-tdTomato deposit or spillover in the hippocampus and/or other subcortical structures. Scale Bars: 500 μm. **(D)** High magnification confocal image of the injection site showing tdTomato expressing neurons. Scale bar: 100 μm. **(E)** Top panel: bright field and epifluorescence image of tdTomato expressing VIP neuron used in whole-cell patch clamp recordings. Bottom panel: example of responses recorded from a VIP neuron using red LED at two different powers. **(F)** Reconstruction of the dendritic arborization of biocytin-filled tdTomato expressing VIP neurons. Scale bar: 100 μm. RSP, retrosplenial cortex; VIS, visual cortex; TeA, temporal association cortex; AC, auditory cortex; HPF, hippocampal formation; CA1, Cornu Ammonis of the hippocampus subfield 1; CA3, Cornu Ammonis of the hippocampus subfield 3; DG, dentate gyrus; TH, thalamus; MGB, medial geniculate body; LGN, lateral geniculate nucleus; MB, midbrain; SN, substantia nigra; HY, hypothalamus.

To better investigate this hypothesis, at the whole-brain scale, we used an anterograde viral tracing approach injecting the right auditory cortex of VIP-Cre driver mice with a Cre-dependent AAV-flex-ChRimson-tdTomato (Figure 2A). This method allowed us to visualize VIP-expressing neurons in the right auditory cortex (Figures 2B–D) and their axonal projection far from the injection site (i.e., long-range). Although AAV1 viruses can display both anterograde and retrograde expression in our experience, this viral preparation exhibits only anterograde features. We also checked every animal injected, and we found no labeled neuron outside the injection site with no evidence of Chrimson-tdTomato deposit or spillover in the hippocampus and/or other subcortical structures (Figure 2C). We also confirmed that tdTomato positive neurons in the injection site (Figure 2D) were expressing ChRimson, patching them while flashing red LED light on the slices to elicit action potentials (Figure 2E). The dendritic arborization of six patched and biocytin-filled neurons was also reconstructed and showed various characteristic somatodendritic features of VIP-like including four bipolar, one modified bipolar (i.e., tripolar), and one multipolar morphology, as previously described (Mesik et al., 2015; Pronneke et al., 2015; Figure 2F).

**Figure 3:**
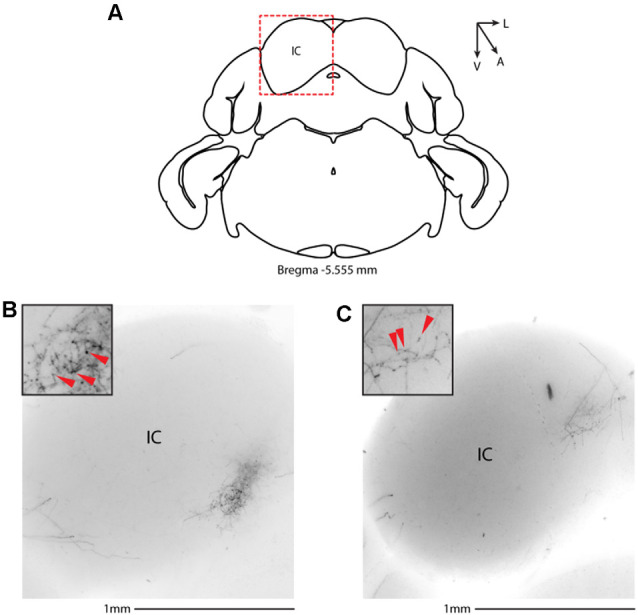
Subcortical target of long-range VIP-expressing neurons: midbrain. **(A)** Allen Brain reference coronal section at −5.555 mm from Bregma. Red dashed squares indicate the region imaged in the panels below. **(B,C)** Two examples of the inferior colliculus, from two animals. Scale bars: 1 mm. Insets indicate axonal branches and the red arrowheads boutons. Inset size 190 μm. IC, inferior colliculus.

**Figure 4:**
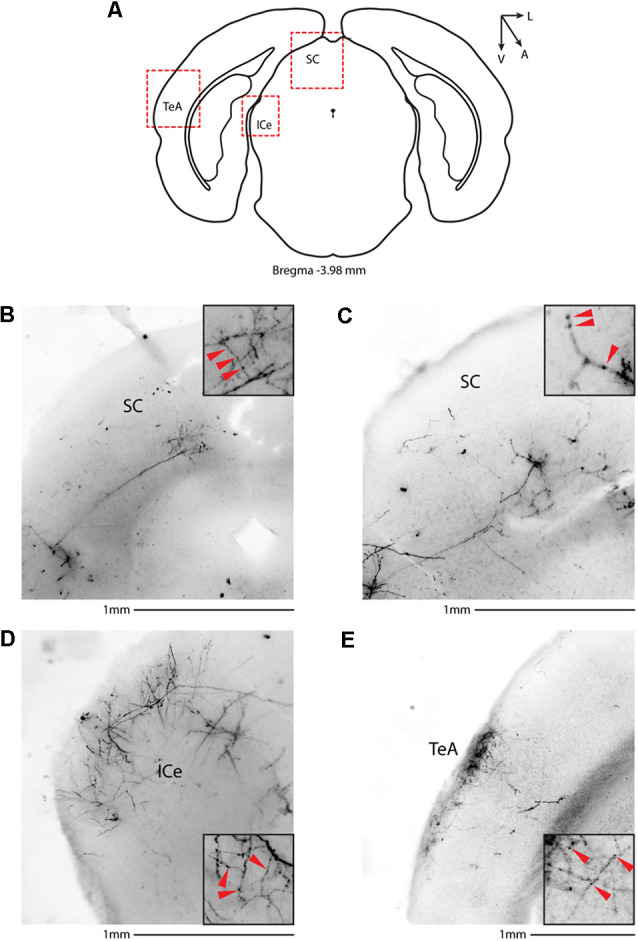
Subcortical target of long-range VIP-expressing neurons: midbrain. **(A)** Allen Brain reference coronal section at −3.98 mm from Bregma. Red dashed squares indicate the regions imaged in the panels below. **(B,C)** Two examples of the superior colliculus, from two animals. **(D)** Representative image of the external cortex of inferior colliculus. **(E)** Representative image of the temporal association cortex. Scale bars: 1 mm. Insets indicate axonal branches and the red arrowheads boutons. Inset size 190 μm. SC, superior colliculus; ICe, inferior colliculus, external; TeA, temporal association cortex.

**Figure 5:**
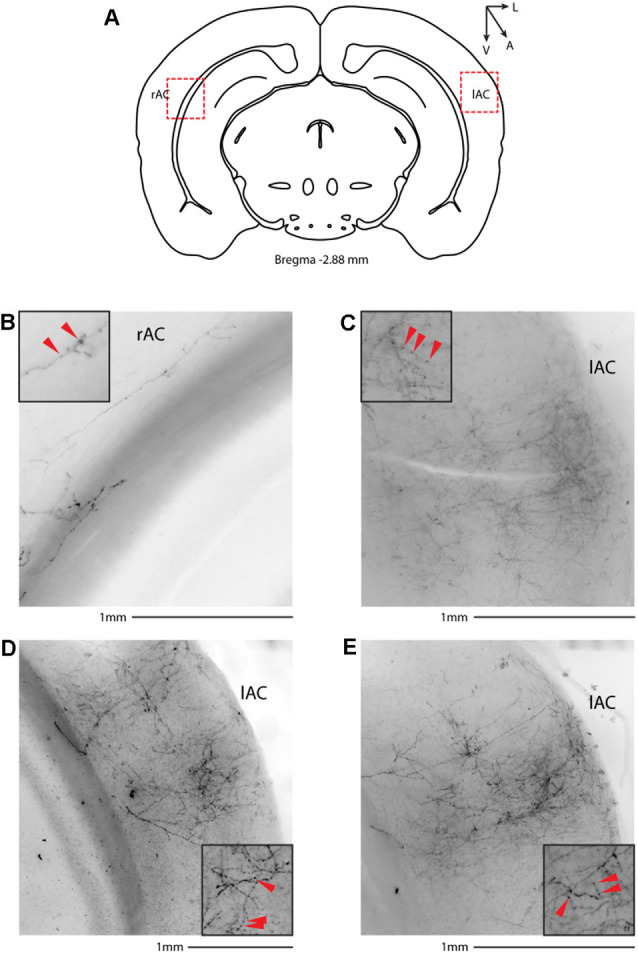
Cortical target of long-range VIP-expressing neurons: auditory cortex. **(A)** Allen Brain reference coronal section at −2.88 mm from Bregma. Red dashed squares indicate the regions imaged in the panels below. **(B)** Representative image of white matter infiltrating axons in correspondence to the injection site. **(C–E)** Three examples of contralateral auditory cortex from two animals. Scale bars: 1 mm. Insets indicate axonal branches and the red arrowheads boutons. Inset size 190 μm. lAC, left auditory cortex; rAC, right auditory cortex.

**Figure 6:**
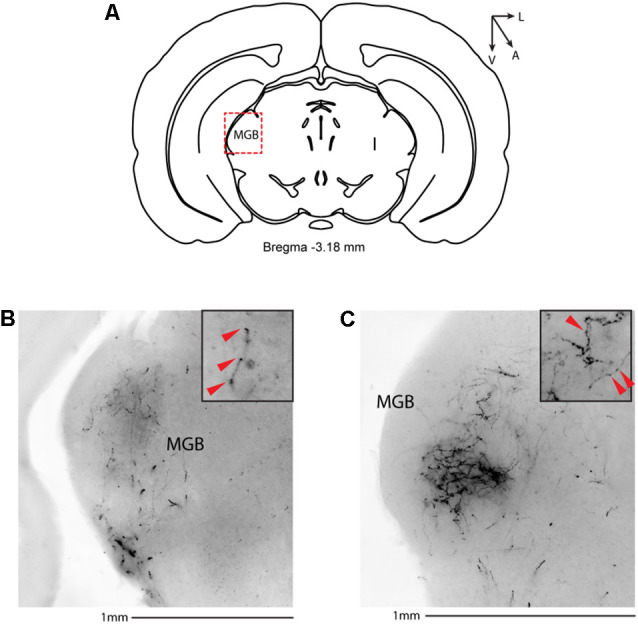
Subcortical target of long-range VIP-expressing neurons: thalamus. **(A)** Allen Brain reference coronal section at −3.18 mm from Bregma. The Red dashed square indicates the region imaged in the panels below. **(B,C)** Two examples of the medial geniculate body from two different animals. Scale bar: 1 mm. Insets indicate axonal branches and the red arrowheads boutons. Inset size 190 μm. MGB, medial geniculate body.

**Figure 7:**
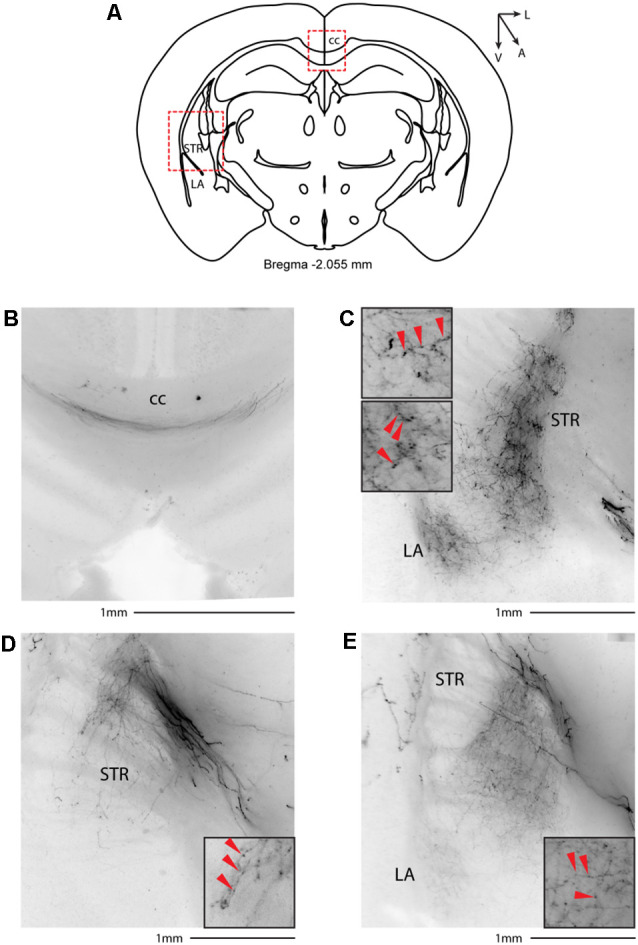
Subcortical target of long-range VIP-expressing neurons: striatum. **(A)** Allen Brain reference coronal section at −2.055 mm from Bregma. Red dashed squares indicate the regions imaged in the panels below. **(B)** Thick axons of passage in the corpus callosum. **(C–E)** Three representative images of VIP axons in the striatum and lateral amygdala from two animals. Scale bars: 1 mm. Insets indicate axonal branches and the red arrowheads boutons. Inset size 190 μm. cc, corpus callosum; STR, striatum; LA, lateral amygdala.

We then systematically imaged every slice, from the most posterior to the most anterior, that displayed VIP-tdTomato axons in *n* = 5 mice from three litters. In midbrain structures, known to be innervated by corticofugal excitatory axons from the auditory cortex (Zurita et al., 2018b), we could observe labeling in the ipsilateral inferior colliculus (Figures 3A–C), superior colliculus (Figures 4A–C), and in the inferior colliculus (Figure 4D) with thick beaded tdTomato+ axons. Moreover, we found a dense arborization of tdTomato+ axons in the temporal association cortex’s upper layer (TeA, Figure 4E). Moving forward on the anteroposterior axis, high magnification images close to the injection site (Figures 5A,B) showed axonal arborization infiltrating the white matter, an indication of potential corticofugal projections. Previous studies from our lab have reported long-range cortico-cortical Parvalbumin-expressing neurons in the auditory cortex that provide local inhibition onto nearby pyramidal neurons and receive thalamocortical input. When looking at the contralateral auditory cortex, we observed a dense tdTomato+ axonal arborization spanning all layers, with a higher concentration in the upper ones (Figures 5C–E), also demonstrating that the VIP-expressing neurons establish cortico-cortical connections. In thalamic areas, we found axons in the medial geniculate body (MGB), the primary auditory thalamus (Figure 6), while in more anterior slices, we found axons in the corpus callosum (Figures 7A,B), and a dense arborization in the striatum and lateral amygdala, spanning at least 600 μm anterior to posterior and almost 1 mm medial to lateral, with a higher concentration of terminal field axons (thin and twisted) in the ventral part of the striatum and thick straight axons of passage in the upper striae (Figures 7C–E).

**Figure 8:**
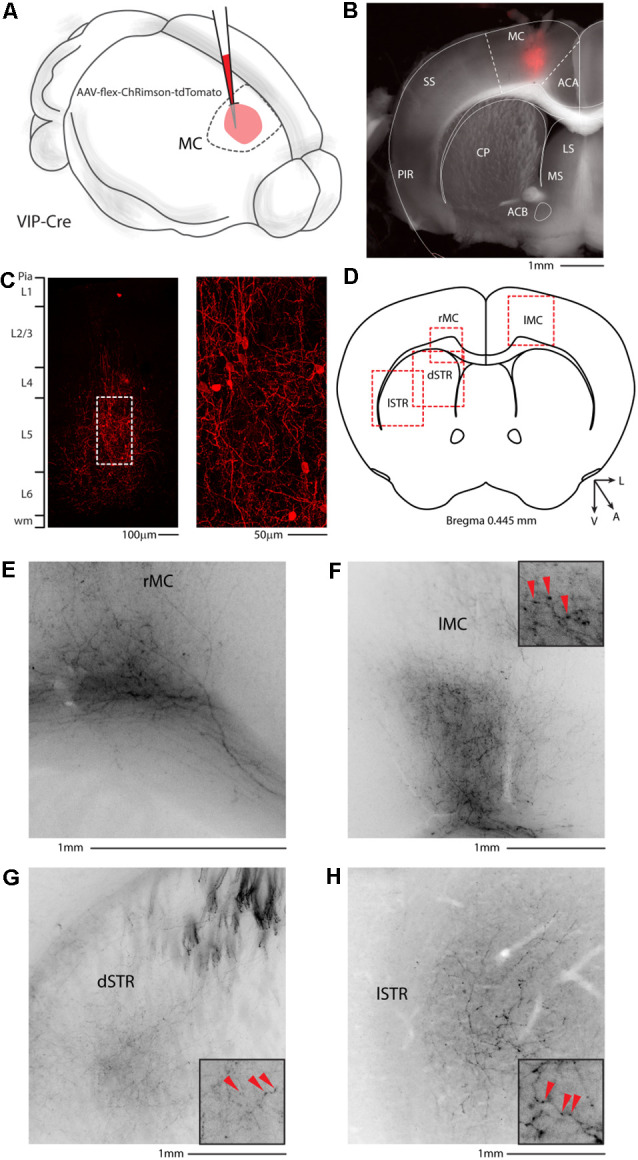
Motor cortex injection of AAV-flex-ChRimson-tdTomato. **(A)** Schematic representation of right motor cortex viral injection. (**B)** Representative coronal section of an acute slice of the injection site (200 μm thick), with bright field (gray) and tdTomato expressing neurons (red). The Allen brain atlas coronal table superimposed for reference indicates the correct targeting of the motor cortex. Scale bar: 1 mm. The image of the injection site showing no evidence of ChRimson-tdTomato deposit or spillover in the striatum and/or other subcortical structures. **(C)** High magnification confocal image of the injection site showing tdTomato expressing neurons. Right panel: high magnification of dashed square in the left panel. Scale bars: 100 and 50 μm. **(D)** Allen Brain reference coronal section at +0.445 mm from Bregma. Red dashed squares indicate the regions imaged in the panels below. **(E)** Representative image of the contralateral motor cortex. **(F)** Representative image of white matter infiltrating axons in correspondence to the injection site. **(G)** Representative image of dorsal motor striatum with thick axons of passage in the upper striae and terminal field axonal arborization in the striatal parenchyma. **(H)** Representative image of lateral motor striatum VIP axons. Scale bars: 1 mm. Insets indicate axonal branches and the red arrowheads boutons. Inset size 190 μm. ACA, anterior cingulate cortex; MC, motor cortex; SS, somatosensory cortex; PIR, piriform cortex; STR, striatum; CP, caudoputamen; ACB, nucleus accumbens; LS, lateral septal nucleus; MS, medial septal nucleus; dSTR, dorsal striatum; lSTR, lateral striatum.

Since our lab and others have already demonstrated that the motor cortex sends long-range Parvalbumin- and Somatostatin-expressing axons to the dorsal striatum (Rock et al., 2016; Melzer et al., 2017), a cortical area involved not only in sensory processing but also in planned and motivated behavior, we asked whether long-range VIP neurons are specific for the auditory cortex or are a common feature of the corticofugal circuit organization. We injected the right motor cortex of VIP-Cre mice with AAV-flex-ChRimson-tdTomato (Figures 8A,B), and we looked for long-range projections. We also checked every animal injected, and we found no labeled neuron outside the injection site with no evidence of Chrimson-tdTomato deposit or spillover in the striatum (Figures 8C,D). Not only we could find axons in the white matter underneath the injection site (Figures 8E,F), and in the striatum, with thick axons of passages in the upper striae (Figure 8G) and twisted mesh of axonal arborization in the ventral and lateral striatum (Figures 8G,H), but also in the contralateral motor cortex (Figure 8F), suggesting that the three major classes of GABAergic neurons are organized in long-range projection to reach the major cortical and subcortical target of the area of interest.

## Discussion

**Figure 9:**
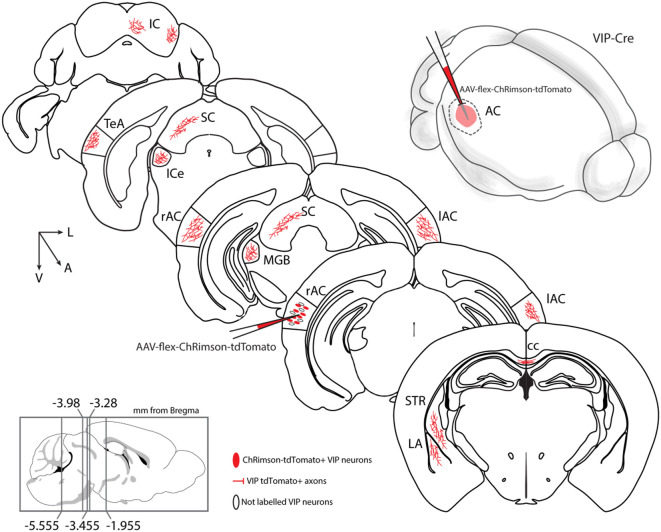
Summary diagram: long-range VIP-expressing neuron projections. Top right: schematic representation of auditory cortex injection with AAV-flex-ChRimson-tdTomato. VIP long-range axons were found in several areas: AC, auditory cortex, right, and left; cc, corpus callosum; ipsilateral ICe, inferior colliculus, external; ipsilateral IC, inferior colliculus; ipsilateral LA, lateral amygdala; ipsilateral MGB, medial geniculate body, ipsilateral SC, superior colliculus; ipsilateral STR, striatum; ipsilateral TeA, temporal association cortex.

In this study, we test the hypothesis that a class of GABAergic neurons, VIP-expressing neurons, establish a long-range GABAergic inhibitory projection in the mouse neocortex. Our results support this hypothesis and additionally conclude that the auditory and motor cortices send long-range GABAergic projections to their corresponding cortex and subcortical structures, such as the dorsal striatum, *via* long-range VIP-expressing neurons (Figure 9). Because of its presence in two such disparate cortical areas, this would suggest that the long-range VIP projection is likely a general feature of the cortex’s network.

A central question of modern neuroscience is determining the whole connectome of the mammalian brain (Abbott et al., 2020) and how many different cell types contribute to signal processing within specific neuronal networks to control behavior. The interaction between two forces, excitation (pyramidal neurons) and inhibition (GABAergic neurons), orchestrate the flow of information within cortical networks. GABAergic neurons are the primary source of inhibition in the adult brain, and they represent a minority of all cortical neurons (10–15% in rodents; Meyer et al., 2011) but are composed of a highly heterogeneous cell population (for review, Ascoli et al., 2008; Xu et al., 2010; Rudy et al., 2011). All our previous knowledge about the cortical cell types has led to a general principle of the organization of the cortical circuit, in which pyramidal neurons are both local and long-range while the GABAergic neurons are only local. However, recent studies from our lab and others (Lee et al., 2014; Rock and Apicella, 2015; Basu et al., 2016; Rock et al., 2016, 2018; Melzer et al., 2017; Zurita et al., 2018a; Bertero et al., 2019, 2020) are demonstrating that long-range GABAergic projections originate from Parvalbumin- and Somatostatin-expressing neurons may be more prevalent than previously assumed.

The present study’s main finding is that the VIP-expressing neuronal population projects through the corpus callosum to connect the two hemispheres of AC and MC, and also reaches subcortical structures such as the dorsal striatum. In addition, our results indicate that the auditory cortex long-range GABAergic VIP-expressing neurons also project to the medial geniculate body, amygdala, temporal association cortex, and superior and inferior colliculus.

Even though we were able to identify long-range VIP-expressing neurons in this study, our approach is limited by caveats (limited injection volume and variability in transfection, which leads to incomplete coverage of the cortical area analyzed) that can preclude us from performing experiments aimed to determine the absolute number and ratio of long-range vs. short–range VIP-expressing neurons. Our findings, together with previous studies, in which long-range GABAergic projections were found to connect different brain areas in different species both ipsi- and contralaterally (Buhl and Singer, 1989; McDonald and Burkhalter, 1993; Tomioka et al., 2005, 2015; Apergis-Schoute et al., 2007; Higo et al., 2007; Tomioka and Rockland, 2007; Tamamaki and Tomioka, 2010; Rock et al., 2016, 2018; Zurita et al., 2018a; Bertero et al., 2019, 2020) lead to a new concept in which not only glutamatergic but also long-range GABAergic cortical neurons are carrying information to far stations (for review, see Isaacson and Scanziani, 2011; Caputi et al., 2013; Melzer and Monyer, 2020). Future experiments will provide insight into the complexity of the long-range VIP GABAergic projections’ anatomical, electrophysiological, and molecular composition.

### VIP neurons: A Disinhibitory Circuits Motif Involved in Synaptic Integration and Plasticity?

In a recent work, Lee et al. (2013) studied a long-range excitatory projection from the motor cortex to the whisker somatosensory cortex. By photo-activating channelrhodopsin-expressing axons originated in the motor cortex and targeting the somatosensory cortex, they found that VIP-expressing neurons received the strongest excitation, compared to Parvalbumin- and Somatostatin-expressing neurons, leading to action potentials only in the VIP-neurons. One of the questions that now needs an answer is if the long-range GABAergic neurons can be recruited by activating long-range associated projection neurons.

Previous work (Dalezios et al., 2002; Pfeffer et al., 2013) has shown that VIP-expressing neurons preferentially inhibit Somatostatin-expressing inhibitory neurons. In line with these findings, recently, Keller et al. (2020) showed that VIP-expressing neurons preferentially inhibit Somatostatin-expressing neurons in the visual cortex, which leads to relief of excitatory neurons from suppression that contributes to contextual modulation in the primary visual cortex. The finding from is similar to what previously Lee et al. (2013) and Pi et al. (2013) observed in the somatosensory and auditory cortex, respectively. Although beyond the target of the present study, it is intriguing to speculate that long-range VIP-expressing neurons could play a role in interhemispheric communication through strong inhibition of Somatostatin-expressing neurons in the contralateral cortex, leading to relief of pyramidal neurons from suppression and eventually entrain the two cortical areas in specific brain oscillations.

In addition, Pi et al. (2013) measured neurons’ activity in the auditory cortex while the mice performed an auditory discrimination behavioral task. In this task, the mice were trained to discriminate two different tones, in which one tone was associated with a water reward and the other with a punishment (Pi et al., 2013). Remarkably, VIP-expressing neurons were strongly activated by reward and punishment signals. However, the strong recruitment of the VIP-expressing neurons did not correlate with an increased spiking of other nearby neurons, as can be expected by the identified canonical cortical disinhibitory circuit motif (for review, see Pfeffer, 2014). One other important aspect of this disinhibitory circuit is that the VIP-expressing neurons, by strongly inhibiting the Somatostatin-expressing neurons, which in turn preferentially inhibit the pyramidal neurons’ distal dendrites (Somogyi et al., 1998), can open a window to increase synaptic integration or plasticity along the distal dendrites of the pyramidal neurons. Are the long-range cortical VIP-expressing neurons part of a long-range disinhibitory circuit leading to integration and plasticity across cortical and subcortical areas? Future experiments will need to address these open questions on the role of the long-range VIP-expressing neurons that can dynamically affect the cortico-cortical processing. On the other hand, it is also intriguing to speculate about the role of long-range VIP-expressing neurons in cortico-subcortical processing: are they establishing a disinhibitory local circuit in their subcortical target, mirroring their main organization in the cortex? For example, in the MGB, only a negligible fraction of the neuronal population express GABA (Lu et al., 2009; Jager et al., 2021) and inhibition has been described to come mainly from the thalamic reticular complex and the inferior colliculus (Rouiller et al., 1985; Winer et al., 1996): would this novel long-range VIP neuron population target the few local GABA-ergic neurons or perform direct inhibition of excitatory neurons in the MGB? A direct source of inhibition from high order auditory stations like the auditory cortex would add a further level of complexity in the modulation of auditory thalamus activity and plasticity not only during perception but also associative learning. Moreover, we showed long range VIP-expressing neurons reaching both the external cortex of the inferior colliculus and the multisensory deeper layers of the superior colliculus, two interconnected midbrain regions involved in orienting behavior (Masullo et al., 2019), suggesting a role for inhibitory long-range VIP-expressing neurons of the auditory cortex in movement selection.

Recent studies from Bigelow et al. (2019), and Yavorska and Wehr (2021) measured movement, and VIP-expressing neurons activation can differentially modulate auditory processing in the mouse auditory cortex. Nevertheless, it is not presently understood whether these differential changes in firing rate, resulting from movement and due to the activation of long-range-GABAergic VIP-expressing neurons, produce corresponding changes in information transfer to the targeted areas. Future studies will provide anatomical, electrophysiological, and molecular features of the long-range VIP-expressing neurons aimed to understand their circuit organization involved in reward-learning and action-selection behavior driven by auditory and motor stimuli.

## Data Availability Statement

The raw data supporting the conclusions of this article will be made available by the authors, without undue reservation.

## Ethics Statement

The animal study was reviewed and approved by IACUC.

## Author Contributions

AjA designed research, performed experiments, analyzed data, and wrote the article. AB performed experiments, analyzed data, and wrote the article. CG performed experiments. All authors contributed to the article and approved the submitted version.

## Conflict of Interest

The authors declare that the research was conducted in the absence of any commercial or financial relationships that could be construed as a potential conflict of interest.

## Publisher’s Note

All claims expressed in this article are solely those of the authors and do not necessarily represent those of their affiliated organizations, or those of the publisher, the editors and the reviewers. Any product that may be evaluated in this article, or claim that may be made by its manufacturer, is not guaranteed or endorsed by the publisher.

## Footnotes

^1^www.ephus.org

## Abbreviations

AC: auditory cortex
ACA: anterior cingulate cortex
ACB: nucleus accumbens
CA1: Cornu Ammonis of the hippocampus subfield 1
CA3: Cornu Ammonis of the hippocampus subfield 3
cc: corpus callosum
CP: caudoputamen
DG: dentate gyrus
ENT: enthorinal cortex
HPF: hippocampal formation
HY: hypothalamus
IC: inferior colliculus
ICe: inferior colliculus; external
LA: lateral amygdala
LGN: lateral geniculate nucleus
LS: lateral septal nucleus
MB: midbrain
MC: motor cortex
MGB: medial geniculate body
MS: medial septal nucleus
NB: nucleus of the brachium of the inferior colliculus
PAG: periacqueductal grey
PG: pontine gray
PIR: piriform cortex
RSP: retrosplenial cortex
SC: superior colliculus
SN: substantia nigra
SS: somatosensory cortex
STR: striatum
TeA: temporal association cortex
TH: thalamus
VIS: visual cortex
VTA: ventrotegmental area.
